# Inhalation anesthesia and shielding devices to allow accurate preclinical irradiation of mice with clinical linac-based systems: Design and dosimetric characteristics

**DOI:** 10.1016/j.ctro.2020.11.012

**Published:** 2020-12-01

**Authors:** Tonny Lagerweij, Charlotte Sewing, Leo van Battum, Phil Koken, Stan Heukelom

**Affiliations:** a Department of Neurosurgery, Cancer Center Amsterdam, Amsterdam University Medical Centers, Location VUmc, Amsterdam, the Netherlands; b Department of Paediatric Oncology, Cancer Center Amsterdam, Amsterdam University Medical Centers, Location VUmc, Amsterdam, the Netherlands; c Department of Radiation Oncology, Amsterdam University Medical Centers, Location VUmc, Amsterdam, the Netherlands

**Keywords:** Animal models, Linac, Inhalation anesthesia, Fractionated radiation, Small animal irradiation devices

## Abstract

This technical note describes two devices to enable accurate irradiation of mice on clinical linac-based systems. To study the effects of radiation in murine, preclinical animal models, controlled and accurate dosing is important. This is not only important when specific volumes need to be irradiated, but also when the whole animal body is irradiated. To enable both purposes, we designed two devices. One device to administer Total Body Irradiation (TBI) simultaneously to six, free walking mice, and a second device, denoted as target box, in which we irradiate specific parts of the mice whilst organs-at-risk (OAR) are protected. In this latter device, we can position the mice in multiple ways. One configuration allows to sedate twelve mice simultaneously by isoflurane inhalation anesthesia and protect the body by lead shielding to allow radiation of the head only. Alternatively, the target box can be used to sedate maximal 4 mice simultaneously to irradiate the flank or paws only. All these setups allow high experimental throughput and thus a minimal occupation of the clinical equipment. As measured, the delivered radiation dosages in the regions of interest were accurate for both devices. In this technical note, we describe the design and build of these devices.

## Highlights

• Up to twelve mice can be treated simultaneously.
• Inhalation anesthesia minimizes discomfort, allowing fractionated treatment protocols.
• The in-house developed devices give accurate radiation doses on the intended areas.

## 1 Introduction

Radiotherapy plays an important role in many cancer treatment protocols. Although radiotherapy has improved patient survival for many tumor types, there is still a need for optimization of therapies to increase radiation efficacy, decrease radiotoxicity, and for the identification of novel radiosensitizers [1], [2]. Preclinical animal studies are essential to evaluate effective drug-radiotherapy combinations, study the radiobiology of tumor tissues, and assist in the development of more effective treatments to control or cure cancer [3], [4]. Due to the small anatomical size of mice, dedicated instruments have been designed. But as these instrument are costly an do not enable radiation of multiple animals at the same time [5], [6], [7], we have explored the use of clinical systems as alternative to irradiate mice. We developed two devices to enable the use of clinical linac systems for irradiation of mice. First, a box for irradiation of specific targets, based on the sedation of animals by inhalation anesthesia. Second, a box for total body irradiation of free walking mice.

### 1.1 Anesthesia and stress

When total-body mice irradiation is desired, e.g. leukemia research, fixation of the mouse is not a prerequisite. Free moving (i.e., in 2 dimensions) of the mice inside an irradiation box is possible with a limited number of mice to avoid mutual aggression, as long as dosimetric requirements are fulfilled. However, when targeting only the area of interest whilst sparing the organs-at-risk (OAR) is desired, immobilization or anesthesia is needed during irradiation, because, amongst many other factors, stress responses influence the outcome of radiation therapy, forced immobilization of awake animals is not advisable [8], [9]. Therefore, we chose to use anesthesia to immobilize the animals. Two main categories of anesthesia can be considered: injection anesthesia or inhalation anesthesia. Although injection anesthesia would be easily applicable because no specific equipment is needed for this, we have chosen to use inhalation anesthesia. The rationale for this is that constant inhalation anesthesia gives greater safety by limiting the risk of under or overdosing and is much more reproducible: all animals recover almost simultaneously within 5 min due to the rapid recovery time [10]. Importantly, this anesthesia is mild enough to allow repeated anesthesia of the mice, needed for repeated -fractionated- radiation regimen.

### 1.2 Animal irradiation and linac systems

Although several small animal irradiation systems are available on the market, most preclinical animal centers do not have access to such dedicated animal radiation systems, whilst clinical linear accelerators are often available nearby or in the same clinic. The reasons why animal radiation systems are not available range from efficacy, financial, and scientific arguments. Concerning efficacy, most dedicated animal systems can handle only one animal at a time and are therefore highly time-consuming, labor-intensive, and cost-intensive. Scientific arguments include that research irradiators often use radiation sources with lower photon energy (range of ~225 kV) or cesium-137 (662 keV) as compared to clinical systems where the photon energy typically range from 6 to 15 MV. As a consequence, with the lower photon energy of the preclinical systems, skin dose is 100% while the homogeneous dose distribution over the target can only be reached if the target is irradiated from several beam directions.

### 1.3 Dosimetric considerations

With clinical radiation systems, the homogeneous dose distribution is guaranteed by the high photon energy. With the target at the isocenter, i.e. at 1 m from the source, single beam dose inhomogeneity (<3%) over a target volume of 1 cm^3^ is not an issue. The same accounts for total body irradiation (TBI) of a mouse when radiated from opposing directions [11].

Dose buildup, as explained below, is an issue to be solved when using high energy (6–15 MV) flattened photon beams. By using a flattening filter in a linac the dose buildup will range from 14% at skin level increasing to 100% at depth d_max_ which is 1.5 and 2.5 cm for 6 and 15 MV, respectively; that means at least half of the thickness of a normal mouse used in studies will not receive the desired radiation dose. To solve this dose buildup aspect we placed a Perspex bar with a thickness of 1.5 cm, equal to the buildup at 6 MV, in beam direction in front of the mouse [12].

If a restricted area – such as the mouse brain – is the target, OAR-irradiation by the primary beam should be avoided by extra shielding of the OAR with lead shielding blocks positioned just above the mouse. Furthermore, phantom scatter, i.e. Compton scattered photons and electrons from the mouse and surrounding materials, should be minimized as much as possible to prevent damage of the OAR. It is a tight balance between photon and electron scatter. We selected 6 MV photon beam to limit the added bar thickness to 1.5 cm polymethylmethacrylate (acrylate) and consequently limit the electron scatter. However, with this relative low photon dose, scatter to the OAR due to photon scatter cannot be completely avoided [12], [13].

In the case of total-body mice irradiation, the OAR shielding is not an issue. Sufficient build-up can be created if all box walls are thick enough which is the case with 2.5 cm acrylate walls. Air gaps between the build-up material and the target area does have an influence on the administered absolute dose, therefore we measured the dose with phantoms which have a comparable size as mice.

## 2 Method and materials

### 2.1 The target box

**Fig. 1:**
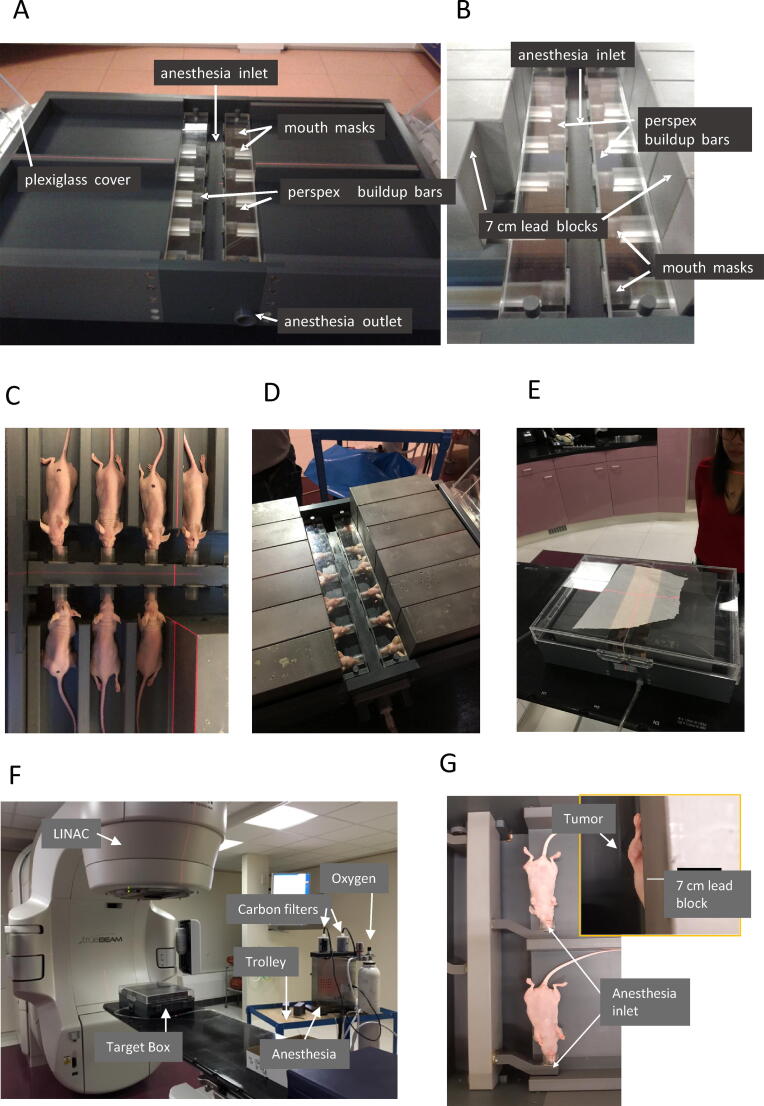
Target Box. Brain targeting configuration (A) Anesthesia gas is delivered through a central anesthesia inlet to 12 mouth masks. Excess anesthesia is removed through the outlet. The head area is covered by acrylate bars to compensate for buildup. The box can be closed with a plexiglass cover. (B) Inlays allow positioning of 7 cm lead blocks to cover the organs-at-risk. (C) Mice are positioned in the mouth masks and (D) covered with 7 cm lead blocks. (E) The closed target box. A piece of paper is put on top of it to enable visualization of the radiation field settings with the X-and Y jaws. (F) The complete setup can be easily transported to the linac. The target box is positioned on the linac bed, whilst the anesthesia remains outside. Mouse Flank configuration (G) With this setup, mice can be positioned such that only one leg/flank will receive radiation.

The design of the radiation device for the mice-brain setting is shown in detail in Fig. 1A–D. Fig. 1A shows a box with 4 compartments in which inlays can be placed to separate the mice. Each compartment contains 3 anesthesia outlets (referred to as masks) where the mice can be positioned with their teeth fixed to an outlet with constant flow of anesthesia gas allowing simultaneously mice-brain irradiations. In Fig. 1B, lead blocks are positioned shielding the OAR-areas whilst the target areas remain uncovered. Fig. 1C, D shows mice fixed in their masks sedated by isoflurane anesthesia. Fig. 1D shows the additional shielding of those mice with a 7 cm extra lead shielding block just above each mouse.

Fig. 1F shows the box and anesthesia device setup on the accelerator. Mice can be anesthetized groupwise on-site just before radiation in a separate anesthesia box, called the induction chamber after which the mice can be transferred to the target box. Anesthesia can be administered by an isoflurane nebulizer (XGI-8 Gas Anesthesia System, Xenogen) both to the induction chamber and the target box. The whole system can be transported easily on a trolley. Except for positioning the mice in their masks, the anesthesia circuit is a closed loop where the excess of anesthesia gas is removed through carbon filters (Fig. 1F). After induction of anesthesia mice are transferred to the irradiation box and positioned in their individual masks (Fig. 1B). Anesthesia masks are available in different sizes to enable accurate positioning of each mouse with respect to the primary beam. The target box is closed during irradiation for optimal control of the anesthesia flow inside the box and prevents contamination of the mice (Fig. 1E).

For a subcutaneous tumor or a tumor in one of the extremities of the animal, an alternative setup of the target box can be configured where the so-called “flank-only inserts” are used. In this configuration the mouse masks are rotated over 90° and the lead blocks are positioned along the body line such that the OARs are protected (Fig. 1G). In this configuration, 4 mice can be treated simultaneously. Different inserts are available to enable left or right-sided irradiation. The further process of anesthesia and positioning of the mice and lead blocks remains the same.

### 2.2 The total body irradiation box

**Fig. 2:**
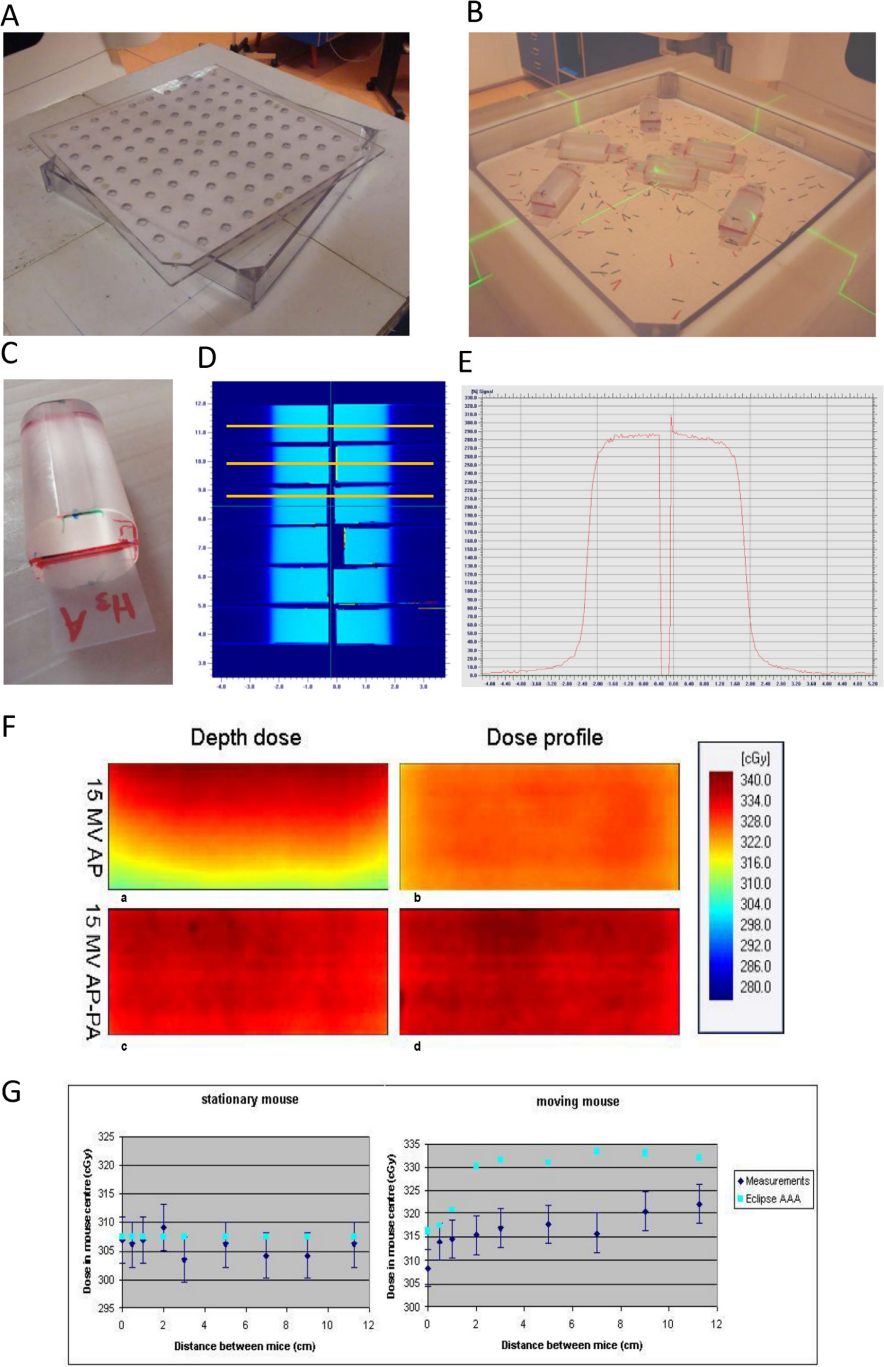
Total Body Irradiation box, phantom design and dosimetry. (A) The acrylate box can be covered by the lid which allows breathing of the mice. Unanesthetized mice can walk freely during radiation. (B) Phantoms are positioned at different positions/orientations (C) Near cylindrical phantoms represent the body of mice. The phantoms are sized 25 × 25 × 50 mm, in which radiochromic film fits in a slit in the middle. (D) Target box: dose profile measurement setup in 12 mouse phantoms covered with 7 cm lead. (E) An example of the dose profile along a yellow line in figure (D) (F) TBI box: 2D-dose distribution for AP and AP-PA treatment technique as measured with EBT film oriented along the photon beam axis. (G) TBI-box: Absolute dose variation as function of distances between mouse phantoms. (For interpretation of the references to color in this figure legend, the reader is referred to the web version of this article.)

For total body irradiation (TBI) of mice, a box inside a box concept is used. The inner box contains the mice (mouse-box) with an inside measure of 30 * 30 * 3.2 cm^3^ (length, width, height), each wall of 0.3 cm thickness. This inside height of this box does not allow the animals to climb on top of each other thus preventing overlapping each other. The lid contains breathing openings with air-filter; the box can also be used for short-time mice transport, allowing sterile transport from the animal facility towards the linac. In case of irradiation, the mice-box is positioned inside the outer box (irradiation-box) exact fitting to the mice-box and with thicknesses of about 2.5 cm for all walls. The breathing openings are covered by the upper (removable) wall of the irradiation box during the beam-on time (Fig. 2A, B).

### 2.3 Control dose measurements

To quantify administered dose in irradiated mice, we made near-cylindrical mouse-like phantoms, sized 25 × 25 × 50 mm, in which radiochromic EBT-3 film fits in a slit in the middle (Fig. 2C). These phantoms were positioned at different locations in the mouse-box simulating the brain-irradiation setting, as well as in the total-body irradiation box using varying settings of those phantoms (Fig. 2B). The film was oriented perpendicular to the primary beam; in addition, the film was oriented parallel to the beam too in the TBI box dosimetry to obtain dose profile in both directions (Fig. 2F). The X- and Y-jaws were set at the desired irradiation geometry [14]. Dosimetric details are given in section C and Appendix.

## 3 Results and discussion

### 3.1 Anesthesia

Athymic nude Foxn1^nu^ mice can be anesthetized by 2–3% isoflurane inhalation at a flow of 1 l/min. Mice are first anesthetized group-wise in the induction chamber after which the individual mice are carefully positioned in the radiation device. At this time, anesthesia can be lowered to 1.5–2.5% isoflurane. Anesthesia induction and positioning of the 12 mice can be done within 5 min. After radiation, mice can be returned to their home cages, and typically they will recover from anesthesia within a couple of minutes.

### 3.2 Dose measurements

#### 3.2.1 Target box

For dosimetry purposes, the exposed films were readout with our standard film dosimetry facility [15], [16]. Dose profiles and absolute dose were read out for six mouse positions (Fig. 2D). Fig. 2E shows a representative dose profile. The observed mean absolute dose is 290 cGy ± 1.7% (1 SD), deduced from the flat profile part. If the tumor is situated at 5 mm from the actual field edge, i.e. set by the additional shielding blocks and defined by the 50% points, it will receive 95% (±1.0% 1 SD) of that absolute dose. Combining the above, a tumor will receive 276 cGy (±2.0% 1 SD) during 300 monitor units (MU) under the condition of the 5 mm distance between target and actual field edge. In practice, that value will be slightly higher (approximately 2%) as targets are positioned in general on top of the mice's skin, i.e. just below the bar, while in our measurement films are positioned more centrally in each mouse phantom. Additional information is given in the Appendix.

#### 3.2.2 TBI box

With respect to dose calculation: Fig. 2G illustrates that radiotherapy dose calculation engines like Eclipse-AAA (Varian, Palo Alto, USA) are not capable to predict the measured outcomes for the moving phantoms. Therefore, in the TBI box administered doses were measured with six mouse phantoms located in the device, in various random settings at distances of 0–11 cm from each other (Fig. 2B). For 6 mouse phantoms present in the box, the absolute dose in the geometrical center of each mice phantom was constant at about 336 cGy ± 1.1% (1 SD) applying an AP-PA irradiation technique. As the absolute dose value might be influenced by the mutual distance of the mice phantoms, this effect was investigated for the AP-irradiation technique by considering the absolute dose with two phantoms: a stationary phantom at the geometrical center of the TBI box and a moving phantom at several distances of that stationary phantom. In the stationary phantom the absolute dose does not change irrespective the position of the other phantom; in the moving phantom an increase up to 5% is observed. (Fig. 2G). The reason is the more near position of that phantom to the acrylate wall of the box with increasing distance from the stationary phantom. Considering this result, a total of six phantoms were positioned randomly and dose measured after applying the AP-PA irradiation technique: a dose increase of 4% was observed in all phantoms, caused by a phantom scatter balance between mutual radiation and shielding of neighboring phantoms. Therefore, in experimental settings the standard number of mice is 6 per box; allowed variation is limited between 4 and 8 mice per box. Experience over many years learns that mice move freely in the box during irradiation, i.e. our experiments seem to reflect real situation.

## 4 Conclusions

Many preclinical radiation experiments are performed with mice. For these experiments, it is important that the dose can be delivered accurately and that the discomfort of the animals is minimized. To enable this on clinically available linac systems, we developed a target box and a TBI box. We quantified that both devices can be used to deliver absolute dosages within an error range of circa 1% (1 SD); and a dose variation of up to 1.5% (1.1% SD) over each mouse. Moreover, the target box setup allowed us to anesthetize with isoflurane, position, and radiate up to 12 mice within 5 min. The target box has already proven its value in the identification of radiosensitizers for the treatment of pediatric brain tumors and glioblastoma [17], [18], whilst the TBI box has been used in several studies, such as those for head-and-neck cancer [19].

In conclusion, the target box allows easy immobilization and positioning of the mice, the mice remain well controlled under anesthesia during the radiations, and both devices support accurate administration of the radiation dose.

## 5 Studies in animals

All animal experiments were done after authorization of the protocols by the local (VUmc animal welfare board) and national (CCD, central committee for animal experiments) authorization boards and all the guidelines have been followed.

## Declaration of Competing Interest

The authors declare that they have no known competing financial interests or personal relationships that could have appeared to influence the work reported in this paper.

## Appendix

**Dosimetric details**

In all experiments, 300 monitor units (MU) were given. 100 MU is defined as 1 Gy dose given in a large water tank at a distance of 100 cm from linac beam focus and at depth d_max_ at the central beam axis of a 10 * 10 cm^2^ at isocenter of the linac [12].

In the target box the side of the lead block just near the tumor is situated at about 5 mm from the tumor and coincides with the field edge of the primary beam at circa 85% dose profile points set by the X-jaws of the accelerator (AP-photon beam, X, Y = 9.6 * 35 cm^2^ with beam axis through the midpoint of the box; (Fig. 2E), 100% = 290 cGy) Just above the mice, i.e. between primary beam focus and mice, an acrylate bar with a thickness of 1.5 cm is placed to create buildup in that bar assuring full dose delivery of the primary beam to the brains (Fig. 1B, D); 6 MV photon beam is used) The beam focus to bar distance is 100 cm. Notice that between and below the head of the mice no additional material is present to limit phantom scatter as much as possible.

Anterior-to-Posterior and Posterior-to-Anterior (AP-PA) irradiation of mice in the TBI box yields over each mouse in all directions a dose variation of 1.5% at most (averaged over 6 mouse phantoms; ±1.1% 1 SD) for the dose at the geometrical center of each mouse phantom. The used field size is 34 * 34 cm^2^, i.e. 2.5 cm irradiation of the sidewalls of the outer box. Used photon beam energy is 15 MV.
